# Evaluation of Antimicrobial Properties of Conventional Poly(Methyl Methacrylate) Denture Base Resin Materials Containing Hydrothermally Synthesised Anatase TiO_2_ Nanotubes against Cariogenic Bacteria and *Candida albicans*

**Published:** 2018

**Authors:** Sahar Abdulrazzaq Naji, Tahereh Sadat Jafarzadeh Kashi, Maryam Pourhajibagher, Marjan Behroozibakhsh, Reza Masaeli, Abbas Bahador

**Affiliations:** a *Foundation of Technical Education, College of Health and Medical Technology, Baghdad, Iraq.*; b *Department of Dental Biomaterials, School of Dentistry, International Campus, Tehran University of Medical Sciences (IC-TUMS), Tehran, Iran.*; c *Department of Dental Biomaterials, School of Dentistry, Tehran University of Medical Sciences, Tehran, Iran. *; d *Dental Research Center, Dentistry Research Institute, Tehran University of Medical Sciences, Tehran, Iran.*; e *Dental Implant Research Center, Dentistry Research Institute, Tehran University of Medical Sciences, Tehran, Iran.*; f *Department of Microbiology, School of Medicine, Tehran University of Medical Sciences, Tehran, Iran.*; g *Research Center for Science and Technology in Medicine, Tehran University of Medical Sciences, Tehran, Iran.*

**Keywords:** Titania nanotubes, Denture base resin, Antimicrobial properties, Biofilm, Cariogenic bacteria, Candida albicans

## Abstract

The purpose of this study was to investigate the antimicrobial properties of a conventional poly methyl methacrylate (PMMA) modified with hydrothermally synthesised titanium dioxide nanotubes (TNTs). Minimum inhibitory concentration (MIC), minimum bactericidal concentration (MBC), and minimum fungicidal concentrations (MFC) for planktonic cells of the TiO_2_ nanotubes solution against *Lactobacillus acidophilus,* *Streptococcus mutans *and *Candida albicans* were determined. The powder of conventional acrylic resin was modified using 2.5% and 5% by weight synthesised titanium dioxide (TiO_2_) nanotubes, and rectangular-shaped specimens (10 mm × 10 mm × 3 mm) were fabricated. The antimicrobial properties of ultraviolet (UV) and non-UV irradiated modified, and non-modified acrylic resins were evaluated using the estimation of planktonic cell count and biofilm formation of the three microorganisms mentioned above. The data were analysed by one-way analysis of variance (ANOVA), followed by a post-hoc Tukey’s test at a significance level of 5%. MIC, for *Streptococcus. mutans*, *Lactobacillus. acidophilus*, and *Candida. albicans*, MBC for *S. mutans* and *L. acidophilus *and MFC for *Candida. albicans* were obtained more than 2100 µg/mL. The results of this study indicated a significant reduction in both planktonic cell count and biofilm formation of modified UV-activated acrylic specimens compared with the control group (*p = *0.00). According to the results of the current study, it can be concluded that PMMA/TiO_2_ nanotube composite can be considered as a promising new material for antimicrobial approaches.

## Introduction

Poly methyl methacrylate (PMMA) acrylic resin is the most commonly used material for fabrication of removable dentures and intraoral maxillofacial prostheses. Its working characteristics, ease of processing, accurate fit, chemical stability in the oral environment, moderate cost and light weight are some of the favourable properties of PMMA that have made it a suitable material for denture base fabrication. Despite these desirable properties, PMMA denture base resin is susceptible to colonisation of microorganisms in the oral environment (1). Surface roughness, porosity, continual denture wearing, and poor denture hygiene are some factors which may have effect of the adhesion of microorganisms and biofilm formation on the surfaces of acrylic resins (2-4).

A wide variety of microorganisms are detected on removable denture base materials, including Gram-positive organisms, Gram-positive and negative rods, as well as Gram-negative cocci and fungi. 

*C. albicans* and *C. glabrata* are the predominant microorganisms that are isolated from denture base acryles (5, 6). *Candida* species can colonise the surfaces of denture base resins and cause biofilm formation, which is the main etiologic agent for the development of denture stomatitis (7, 8). Moreover, the surface of acrylic denture base in a removable partial denture or orthodontic appliance encourages the residence and accumulation of aerobic and anaerobic, and also facultative micro flora such as streptococci and lactobacilli, which are the main cause of dental caries. Therefore, different efforts have focused on developing an antimicrobial denture base resin. Methallyl phosphate monomers (9), methacrylic acid monomers (10), 2-tert-butylaminoethyl methacrylate (TBAEMA) (11), silver zeolites (12, 13), silver nanoparticles (AgNPs) (14, 15), and titanium oxide nanoparticles (TiO_2_NPs) (16) are some antimicrobial agents which have been used for production of an antimicrobial acrylic denture base.

Titanium dioxide nanoparticles have strong antimicrobial activity through photocatalysis. It has been reported that the anatase crystalline form of titanium dioxide (TiO_2_) displays photocatalytic activity under ultraviolet A (UVA) illumination. The irradiation of UVA light with a wavelength less than 385 nm can activate crystalline TiO_2_ and generate electron (e^-^)-hole (h^+^) pairs. The excited electrons can react with electron acceptors like oxygen and produce a superoxide ion (O_2_^•-^). The positive holes also can react with H_2_O or OH^-^ and generate hydroxyl radicals (•OH). Other reactive oxygen species (ROS), like hydrogen peroxide (H_2_O_2_) may also be produced. The reactive oxygen species including (O2•-), (•OH), and H_2_O_2_ can decompose nearby organic compounds(17-19). The photodecomposition property of TiO_2_ has been investigated for employment in various antimicrobial approaches (20-22). The mechanism results from the reaction between reactive oxygen species and the outer membrane of bacteria, like cell membrane or cell wall, which leads to leakage or damage of the cell (23). Different studies have demonstrated the death of Gram-positive and Gram-negative bacteria and fungi like *Streptococcus mutans*, *Escherichia coli*, and *C. albicans *by the photocatalysis of TiO_2 _(1, 24 and 25).

Recently, nanostructured titania has been widely used and synthesized via different methods as a photocatalyst due to its higher photocatalytic activity than mico-scaled titania (26). The higher photocatalytic activity mainly results from the high specific surface area of the TiO_2_ nanostructure. Different studies have added TiO_2_ nanoparticles to dental restorative materials and demonstrated antibacterial and improved mechanical properties. In this regard, Anehosur *et al*. (27) modified the poly methyl methacrylate denture base resin using titania nanoparticles and found inhibitory activity against *S. aureus *in the fabricated PMMA/TiO_2_ nano-composites. They suggested that PMMA denture base polymer modified using light activated TiO_2_ NPs could improve the dental hygiene of denture wearers.

Sodagar *et al.* (28) also reported strong antimicrobial activity against the cariogenic bacteria, including *L. acidophilus* and *S. mutans *in PMMA acrylic resins containing TiO_2_ and SiO_2_ nanoparticles. They also determined that this antibacterial property is more efficient under irradiation of UVA due to the photocatalytic properties of nano-TiO_2_.

Regarding the high specific surface area of nanoparticles, the cylindrical morphology of nanotubes with a hollow cavity at their centre may present increased active surface area compared with nanoparticles and enhance light trapping (29), which ultimately leads to higher antimicrobial activity than that of nanoparticles.

Therefore, this study aimed to investigate the incorporation of TiO_2_ nanotubes on the antimicrobial properties and biofilm formation of a commercial conventional denture base resin. The null hypothesis is that the addition of TiO_2_ nanotubes into denture base polymer does not affect its antimicrobial and biofilm formation properties.

## Experimental

*Study groups*

Conventional acrylic resin (mega CRYL HOT, megadental GMBH, Germany) was modified using titanium dioxide (TiO_2_) nanotubes, which were synthesised and characterised according to our previous study (30). TiO_2_ nanotubes were prepared via an alkaline hydrothermal process from a commercial TiO_2_ nanoparticle powder (SkySpring, Nanomaterials, Inc., 2935 Westhollow Drive, Houston, TX 77082, USA) with a crystalline structure of approximately 99.5% anatase and a particle size of 10–30 nm. The synthesizing procedure of TiO_2_ nanotubes was started by treating 1.14 g of nanoparticle powder with 40-45 mL of 10 N NaOH solution. The suspension then was sealed in a Teflon-lined autoclave at temperatures of 150 °C for 48 h. Subsequently, the resultant precipitates were washed with deionized water and HCl aqueous solution (1 M). Finally, the powders were dried using an oven at 80 °C for 3 h to give the as-synthesized nanotubes. The synthesized TiO_2_ nanotubes and the powder of conventional denture base resin were weighted using an analytical balance (Sartorius, Goettingen, Germany) and then divided into equal parts. Each part of TiO_2_ nanotube powder was mixed manually with powder of acrylic resin. Subsequent to hand mixing they mixed with a dental amalgamator device (Ultramat 2, SDI, Australia) for better particle distribution. The fabricated specimens were assigned to three groups according to the percentage of added TiO_2_ nanotubes including TNT 0% (control), TNT 2.5% and TNT 5% by weight. The antimicrobial and anti-biofilm formation activities of PMMA/TiO_2_ composites were evaluated in both non-UV-irradiated and UV-irradiated samples for three microbial strains including *C. albicans*, *L. acidophilus* and *S. mutans.*

*Sample preparation*

Rectangular-shaped specimens with dimensions of 10 mm × 10 mm × 3 mm were fabricated according to the manufacturer’s instructions and ISO 20795-1:2013 (31). The powder of acrylic resin was modified using 2.5% and 5% by weight synthesized TiO_2_ nanotubes. The proportioned polymer/monomer was mixed and then packed in dental stone moulds (Hydrocal dental stone, Moldano, Bayer Lerekusen, Germany). The two portions of the flask were closed together tightly and pressed slowly at 40,000 N under the hydraulic press so that the dough resin evenly flowed all over the mould space. Then the pressure was released, the two portions of the flask were opened, and the excess material was removed using a sharp scalpel. Finally, the two portions of the flask were closed and placed under a press (20 bars) for 5 min. Subsequently, the flask was left under low pressure for 30 min and then maintained in a water bath at room temperature. The temperature was raised up to 73 ± 1 °C slowly and then was held at the boiling point at 100 °C for 30 min. Finally, the fabricated specimens were subjected to polishing and finishing procedures to obtain to a glossy and smooth surface. Finally, the fabricated samples were sterilised by gamma rays at a dose of 25 kilograys.

*Microbial strains and growth conditions*

*C. albicans* ATCC 90028, *L. acidophilus* ATCC 4356 and *S. mutans *ATCC 25175 were obtained from the Iranian Biological Resource Center (Tehran, Iran) and employed in this study. *S. mutans* and *L. acidophilus* were grown in microaerophilic and anaerobic conditions, respectively, in Brain-Heart Infusion (BHI) broth (Difco, Sparks, MD, USA) at 37 °C until the cells attained the mid-logarithmic phase (OD 600_nm_ = 0.2 for* S. mutans* and OD 600_nm_ = 1.0 for* L. acidophilus*) (32, 33). *C. albicans* strain was cultured on the Yeast Extract Peptone Dextrose (YEPD) broth (10 g yeast extract, 20 g peptone, 20 g dextrose, 1,000 mL distilled water, pH 7.0). The cells of *C. albicans *grew aerobically until they reached the mid-logarithmic growth phase (OD 600_nm_ = 1.0) (34).

*MIC, MBC, and MFC*

The antimicrobial activity of the TiO_2_ nanotube solution was evaluated by measurement of MICs, MBC, and MFC against planktonic microbial cells, as recommended by the Clinical and Laboratory Standards Institute (CLSI) and International Organisation for Standardisation (ISO) (35-37). In this method, a single 96-well sterile polystyrene microtiter plate was used for each microbial strain. A susceptibility panel in the microtiter plates was prepared by pipetting 100 μL of 2 × BHI broth to each well; 100 μL of TiO_2_ solution (10 mg mL^-1^) was added to the wells in column 1 (far left of the plate), and the TiO_2_ concentration was diluted to 1:2 (*i.e.* 5 mg mL^-1^). TiO_2_ was diluted 2-fold by transferring 100 μL aliquots from column 1 to column 2. Therefore, column 2 is a 2-fold dilution of column 1 (*i.e.* 2.5 mg mL^-1^). The process was continued across the microplate to column 10, and then 100 μL was discarded from column 10 rather than dispensing it into column 11. Starting from column 11 to column 1, the columns were inoculated with fresh BHI microbial cultures (100 μL/well) and adjusted to a concentration of 1.0 × 10^6^ CFU/mL for bacterial suspensions and 1.0 × 10^5^ CFU/mL for the *C. albicans* suspension using a multi-channel pipet. In the susceptibility panel, column 11 served as the positive (growth) control, and column 12 was not inoculated and considered as the sterility control.

The MIC was defined as the lowest concentration (μg mL^-1^) of TiO_2_ that inhibited the visible growth of microorganisms. In this regard, after an incubation period, the MIC value was estimated by visual examination. The MBC and MFC determined the lowest concentration of TiO_2_ to kill tested bacteria or fungi, respectively. The MBC and MFC were then found by subculturing (10 μL) the contents of each well without visible growth onto BHI agar plates. After 24 h of incubation of BHI agar plates at 37 °C, the colony-forming units per millilitre (CFU mL^-1^) were determined using the Miles and Misra Method (38). The MBC and MFC were thus determined as the lowest concentration (μg mL^-1^) of TiO_2_ yielding ≥99.9% reduction of the initial CFU mL^-1^ after incubation.

*Planktonic growth assay*

The antibacterial and antifungal activities of non-UV- and UV-irradiated TiO_2_ nanotubes were evaluated in the three aforementioned groups including control (TNT 0%), TNT 2.5%, and TNT 5% (n = 15) via estimation of the planktonic phase for each mentioned microbial strain separately.

In this assay 1.5 × 10 ^5^ CFU mL^-1^ of freshly prepared microbial suspensions were poured into 2 mL tubes, and then the prepared acrylic samples were placed in the tubes containing microbial suspensions.

To treat the PMMA/TiO_2_ nanotube samples with UV irradiation, the acrylic disks were placed in a chamber equipped with a 15 W BLB lamp (Philips Electronics, Seoul, Korea), and the emitting radiation was at 350–410 nm. The distance between the lamp and the acrylic samples in an anaerobic cabinet was set up to obtain 1.0 mW/cm^2^ of ultraviolet type A (UVA) incident light. UVA light was emitted for 10 min, and the intensity of UV light was measured by a UVA radiometer (Konica Minolta) (39, 40).

After UV irradiation, 10 μL aliquots of tube suspensions containing microorganisms and PMMA-TiO_2_ nanotube composites were inoculated into a flat-bottom, polystyrene 96-well microtiter plate, of which each well had previously been prepared to a volume of 90 μL with BHI broth. A serial dilution (10^-1^, 10 ^-2^, 10^-3^, 10 ^-4^ and 10^-5^ dilutions) was then performed, and 10 μL from each well was inoculated in the BHI agar. Subsequently, a spread culture procedure was done and incubated according to the incubation conditions of the above-mentioned strains for 24 h at 37 °C, and the count of vital bacteria and fungi was determined as CFU mL^-1^ following incubation as mentioned above.

**Table 1:** Viable planktonic phase counts of *C. albicans, L. acidophilus *and *S. mutans *in non-modified conventional denture base acrylic discs (control group) and those modified with 2.5% and 5% TiO2 before and after UV irradiation

**Microbial strains (CFU mL** **-1** **) (mean ± SD)**							
Groups	**Non UV irradiated samples**			**UV irradiated samples**			
	***C. albicans***	***L. acidophilus***	***S. mutans***	***C. albicans***	***L. acidophilus***	***S. mutans***
Control	10.48 ± 0.013	10.28 ± 0.004	10.27 ± 009	8.27 ± 0.012^a^	8.24 ± 0.003	8.15 ± 0.010^c^
TiO2-2.5%	8.23 ± 0.016^a^	8.14 ± 0.126	8.07 ± 0.013^b^^c^	6.62 ± 0.029	6.43 ± 0.030	6.16 ± 0.079
TiO2-5%	8.05 ± 0.027	8.03 ± 0.008	8.02 ± 0.014^b^	5.09 ± 0.081	4.90 ± 0.054	4.77 ± 0.082

The same letters denote the groups which have statistically significant differences.

a *p *= 0.5 for the difference between the microbial count of non-UV-irradiated TiO -2.5% and UV irradiated control samples of *C. albicans.*

b *p *= 0.610 for the difference between non-UV TiO -2.5% and TiO -5% samples of *S. mutans.*

c *p = *0.145 for the difference between non-UV TiO -2.5% and UV-irradiated control samples of *S. mutans*.

**Table 2:** Viable cell counts of *C. albicans, L. acidophilus *and *S. mutans *biofilms in non-UV activated resin and following activation with UV irradiation in a conventional denture base acrylic resin containing 0% (control), 2.5% and 5% titania nanotubes

**Microbial strains (CFU mL** **-1** **) (mean ± SD)**						
Groups	**Non UV irradiated samples**			**UV irradiated samples**		
	***C. albicans***	***L. acidophilus***	***S. mutans***	***C. albicans***	***L. acidophilus***	***S. mutans***
Control	6.43 ± 0.020^a^	6.36 ± 0.036^c^^d^	6.20 ± 0.020^f^^g^	6.33 ± 0.057	6.28 ± 0.036^d^	6.17 ± 0.024^g^
TiO2-2.5%	6.36 ± 0.016^a^	6.27 ± 0.020^c^	6.13 ± 0.013^f^	6.14 ± 0.027	6.02 ± 0.017^e^	5.86 ± 0.076
TiO2-5%	6.18 ± .040^b^	6.07 ± 0.034^e^	5.99 ± 0.038	4.86 ± 0.083^b^	4.46 ± 0.141	4.39 ± 0.080

The same letters denote the groups which have not statistically significant differences.

*albicans.*

*acidophilus.*

a *p *= 0.133 for the difference between biofilm formation on non-UV-irradiated TiO -2.5% and control samples of *C. albicans.*

b *p *= 0.706 for the difference between biofilm formation on non-UV-irradiated TiO -5% and UV irradiated samples of TiO -5% of *C.*

c *p *= 0.341 for the difference between biofilm formation on non-UV-irradiated TiO -2.5% and control samples of *L. acidophilus*.

d *p *= 0.497 for the difference between biofilm formation on non-UV-irradiated and UV irradiated control samples of *L. acidophilus*.

e *p *= 0.746 for the difference between biofilm formation on non-UV-irradiated TiO 5% and UV irradiated TiO -2.5% samples of *L.*

f *p *= 0.248 for the difference between biofilm formation on non-UV-irradiated TiO -2.5% and control samples of *S. mutans.*

g *p *= 0.956 for the difference between biofilm formation on non-UV-irradiated and UV irradiated control samples of *S. mutans*.

**Figure 1:**
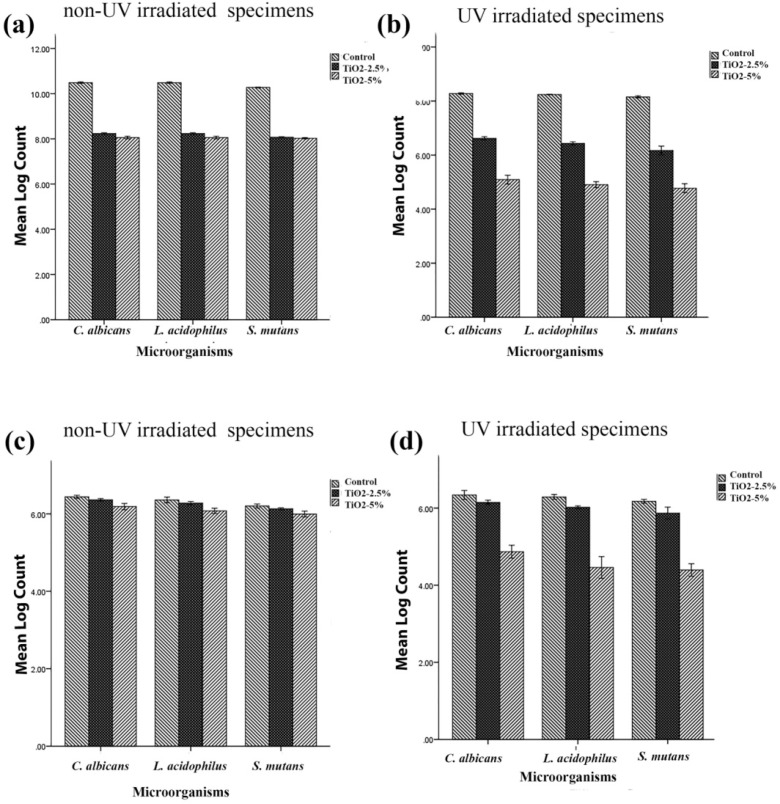
Viable microorganism counts (CFU mL^-1^) of *C. albicans, L. acidophilus* and *S. mutans* in (a) planktonic phase assay of non-UV activated samples, (b) planktonic phase assay following activation with UV irradiation, (c) biofilm of non-UV irradiated samples and (d) and biofilm following activation using UV irradiation. (Error bars: +/-2 SD)

**Figure 2:**
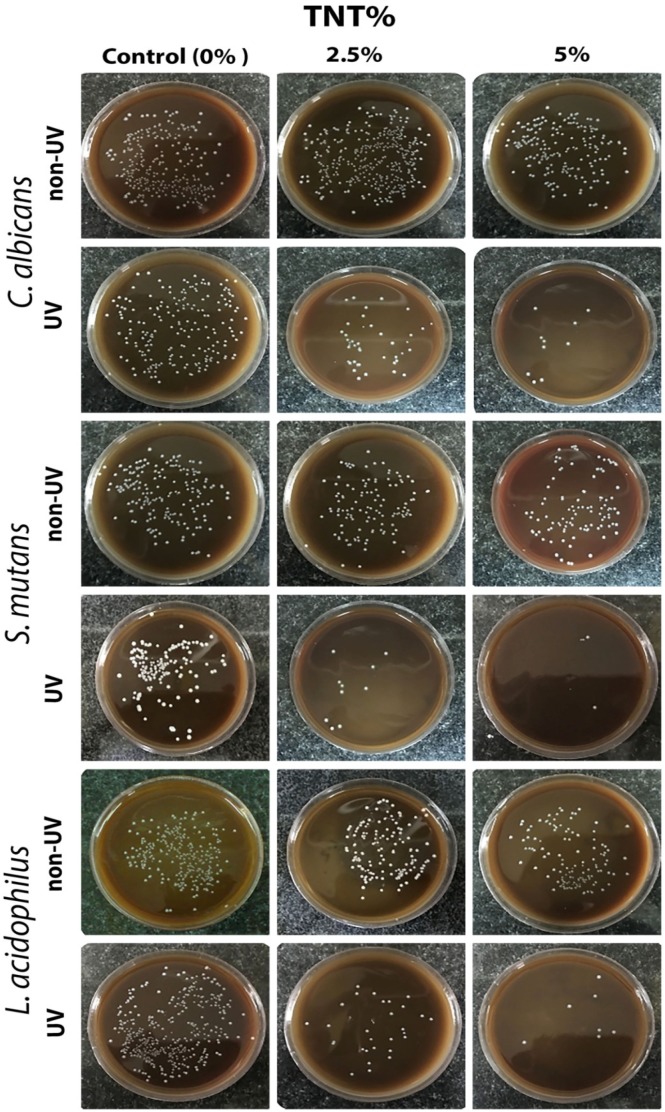
Representative image of the microbial count of *C. albicans*, *L. acidophilus *and *S. mutans *evaluated by planktonic phase assay in non-UV- and UV-irradiated specimens. As it is shown, the number of microorganisms was reduced in TiO2 nanotube (TNT)-activated samples in all utilised strains

*Biofilm formation*

The biofilm formation on the surface of the acrylic samples placed in the tubes containing three aforementioned microbial suspensions was evaluated separately for each strain before and after UV irradiation. Then, the microorganisms were incubated for 48 h at 37 °C under the proper incubation conditions for each strain. After incubation, the specimens were gently washed twice with 3 mL sterile phosphate-buffered saline (PBS) (10 mM Na_2_HPO_4_, 2 mM NaH_2_PO_4_, 2.7 mM KCl, 137 mM NaCl, pH 7.4) to remove the nonadherent and loosely bound cells. After that, the specimens were placed in the tubes containing 1 mL of BHI broth and sonicated using a sonicator (Branson, China) with a frequency of 50 Hz and 150 W power for 5 min. Serial dilutions were performed as described in the previous section. Ten microlitres from each diluted microbial suspension was transferred to BHI agar medium and spread over the entire agar surface with a sterile spreader. Following incubation for 24 h at 37 °C, the viable bacteria were counted and their number were calculated as CFU disk^-1^ as mentioned above.

*Statistical analysis*

The homogeneity and normality of variances of the data were tested before statistical analysis. The normality of data was investigated and confirmed by Kolmogorov-Smirnov analysis at a significance level of 5%. 

Then, the data were analysed by one-way analysis of variance (ANOVA), followed by a post hoc Tukey’s test at a level of significance of *p* < 0.05. The data were analysed using SPSS 23.0 for windows.

## Results

In the current study, the growth rate of planktonic cells and biofilm formation of three microbial strains including *C. albicans, L. acidophilus and S. mutans* were evaluated for the non-UV and UV-irradiated conventional and titania nanotube-modified denture base acrylic resin.

*MIC, MBC and MFC*

The MIC, for *S. mutans*, *L. acidophilus* and *C. albicans, *MBC for *S. mutans* and *L. acidophilus* and MFC for *C. albicans* obtained were greater than 2100 µg/mL.

*Planktonic growth phase assay*

Table 1 and Figures 1a and 1b show the antimicrobial effect of three non-irradiated groups of fabricated samples including non-modified acryles and those modified with 2.5% and 5% titania nanotubes against the three above-mentioned microbial strains. Although the UV- irradiated disks exhibited a more efficient antibacterial effect than the non-UV irradiated disks for three microbial strains (*p = *0.00), the non-UV irradiated samples also showed a significant reduction in the microbial count in both the 2.5% and 5% TiO_2_ nanotube-modified groups (*p = *0.00).

The results also demonstrated that though the activation of TiO_2_ nanotubes in the control group did not affect biofilm formation in any of the three microbial strains (TNT 0%), in the planktonic growth assay, the microbial count of the control group was significantly reduced in UV-irradiated samples in comparison with non-UV irradiated ones (*p = *0.00).

The representative images of the microbial viability of the three different microbial strains employed in non-UV- and UV-irradiated acrylic samples are shown in Figure 2.

*Biofilm formation*

The formation of mature biofilms on the surface of three different acrylic resins including nanotubes modified with 2.5% and 5% titania, and the control group (TNT, 0%) were recorded in the current study. Counts of viable bacteria and fungi are shown in Table 2 and Figures 1c and 1d. One-way analysis of variance showed a significant reduction in biofilm formation of TNT’s modified acrylic resins compared with the control group. Tukey’s post-hoc test indicated a significant reduction of biofilm formation by addition of 5% titania nanotubes to denture base acrylic resins in both non-UV and UV-irradiated samples for three microbial strains (*p = *0.00). Although the addition of 2.5% non-activated TNTs to acrylic resins reduced the viable microbial count, the differences were not statistically significant for *C. albicans *(*p = *0.133) and* L. acidophilus *(*p = *0.341). According to the results of this study, the efficiency of incorporation of 5% non-activated TiO_2_ nanotubes to acrylic resins was not significantly different from that of 2.5% activated TNTs in *C. albicans *(*p = *0.706) and* L. acidophilus *(*p = *0.746). The results also indicated that UV irradiation did not affect biofilm formation on the surface of non-modified acryles in any of the three microbial strains (*p > *0.05).

## Discussion

Poly methyl methacrylate is an ideal material for denture base fabrication due to its desirable properties. However, this material is susceptible to colonisation by various microbial species, including *C. albicans*, *C. glabrata* and gram positive/negative organisms. Different attempts have been made to overcome this drawback of denture base resins. The incorporation of biocide additives like silver zeolites, silver nanoparticles (AgNPs) and titania nanoparticles into the polymer matrix is an approach to developing a denture base acrylic resin with antimicrobial potential (1). In this study a hydrothermally synthesised titania nanotube was incorporated into denture base acrylic resin to improve its antibactrial properties. To the best of our knowledge, this is the first study which incorporated titania nanotubes into the matrix of denture base resin. In our unpublished article, we described the effect of TiO_2_ nanotubes on the mechanical properties, and in the current study, we focused on the antibacterial properties of titania nanotubes.

Since 1972, when Fujishima and Honda first discovered the photocatalytic water-splitting potential of TiO_2_ electrodes (41), their use in environmental applications has gained increasing interest. Besides, its photocatalyst property, TiO_2_ has high chemical stability and is relatively inexpensive (42). Recently, studies have been extended to investigate the microbicidal potential of titania-based photocatalysts against pathogenic microbes, including Gram-positive and negative bacteria, fungi and viruses (43-46). Different studies described the photocatalytic responses of TiO_2_ to ultraviolet (UV) light. In a photocatalysis system under an UV source like sunlight or an artificial ultraviolet source, the electron of the photocatalyst becomes excited and the extra energy of this exited electron creates electron (e-)-hole (h+) pairs. The generated electrons and holes react with water and oxygen and produce reactive oxygen species (ROS), such as (O2•-), (•OH) and H_2_O_2_, which can decompose nearby organic compounds (43). In this study we evaluated the antimicrobial properties of TiO_2_ nanotube-modified acrylic resin against three microbial strains including *C. albicans*, *L. acidophilus,* and *S. mutans *under ambient light and UV irradiation. *C. albicans *is the predominant microorganism isolated from dentures and the main cause of stomatitis in denture wearers. Streptococci and lactobacilli are some of the bacteria which are known as the main cause of dental caries (47, 48). On the other hand, in recent years the demand for orthodontic services and thus the need to use acrylic removable appliances and retainers has increased*. *These removable appliances can promote the plaque accumulation and increase the risk of dental caries (49). The synthesised TiO_2_ nanotubes used in this study showed strong antibacterial and antifungal properties against all employed species, and the MIC, MBC, and MFC values were more than 2100 µg/mL. Earlier we described that the production of reactive oxygen species (ROS) such as •OH, •O_2_−, •HO_2_ and H_2_O_2_ due to the photocatalytic properties of titania leads to the decomposition of microorganisms. Moreover, the attachment of the TiO_2_ nanotubes to the cell membrane of species may affect and upset the permeability of the cells, induce oxidative stresses, and inhibit cell growth (29). Maness *et al.* proposed that ROS generated on the surface of UV-irradiated TiO_2_ can cause lipid peroxidation reactions and subsequently a breakdown in the structure of the cell membrane (18). They considered this function to be the principal mechanism for cell death in *E. coli*. They determined that because all cell membranes are made up of different lipids with various degrees of unsaturation, the proposed cytotoxic mechanism can be extended to all cell types. Kubacka *et al*. described that rapid inactivation of the cells at the regulatory and signalling levels, a strong decrease in the coenzyme-independent respiratory chains, a lower capacity for iron and phosphorous transportation, a lower capacity for the biosynthesis and degradation of heme (Fe-S cluster) groups and wall modifications are the main factors responsible for the high biocidal activity of titania-based nanomaterials (50). Moreover, the higher surface area of nanotubes compared with nanoparticles may lead to greater antibacterial properties. However, Verran *et al.* reported that the antibacterial efficiency of TiO_2_ nanoparticles are more sensitive to the crystallinity of structure than to surface area (51). They reported that the inherent ability of nanoparticles to produce radicals would affect their antibacterial properties.

The results of our study showed a significant reduction in both biofilm formation and viable bacterial count after UV irradiation in PMMA/TiO_2_ nanotubes composites. The TiO_2_ photocatalytic response to UV irradiation is the explanation for the antibacterial properties of UV-irradiated samples. The vital organism count in the control group (TiO_2_ 0%) also showed a significant reduction in UV-irradiated samples compared with non UV-irradiated ones. The reduction of microbial strains in the control group in UV-irradiated samples can be explained by the following mechanisms. Ionising and non-ionising radiation are two forms of electromagnetic radiation. UV light is a non-ionising radiation that exerts its mutagenic effect by exciting electrons in DNA molecules. This excitation leads to the formation of extra bonds between two adjacent pyrimidines in DNA. These bonded pyrimidines form a structure which is called a pyrimidine dimer. The shape of the DNA often changes due to the formation of these dimers, which can cause problems during replication. In this way, UV irradiation can inactivate microorganisms and control microbial growth (52, 53).

In our study, the acrylic samples which had been modified with 5% nanotubes showed greater antibacterial and antibiofilm formation properties than 2.5%-modified and non-modified samples. The higher concentration of TiO_2_ nanotubes may lead to higher ROS and cause greater antibacterial properties. However, Verran *et al.* indicated that in a liquid system high concentrations and aggregates of particles results in fewer antibacterial properties because less light can pass through the suspension (51). In our attempt, greater antibacterial properties were achieved in the samples which were modified using a 5% concentration of TiO_2_, in spite of higher agglomeration of nanotubes.

For synthesis of TiO_2_ nanotubes, we employed the anatase phase of TiO_2_ nanoparticles. The crystalline structure of the material has an important role in its antimicrobial properties. In this regard, different studies determined improved antimicrobial properties for the anatase and rutile crystalline phase of titania (54). Li *et al*. determined the highest antibacterial activity for the anatase nanotubes among three crystalline phases of titania including anatase, rutile, and amorphous (55). Del Curto *et al.* reported a significant reduction in bacterial adhesion and colonisation of *S. mutans*, *S. salivarius*, and *S. sanguis *on anatase-coated titanium of dental implant abutments (56). Surface modification of Ti and surface topography, like the diameter of nanotubes, are other factors that can affect the antimicrobial properties of material (54, 55). Studies also reported that resistance of bacteria to ROS attack affects the antibacterial properties of a material. The ability of a bacterium to tolerate ROS attack depends on its inherent characteristics, such as the cell wall, the thickness and structure of the cell membrane, and ROS-scavenging systems (17, 20). However, all microbial strains utilised for this work showed very similar behaviour in all groups. In photocatalytic disinfection systems, when a large amount of ROS are produced, the excess of ROS would likely overwhelm the ROS scavenging systems of the microorganism (20). It should also be noted that the adsorption of bacteria on titania surfaces affects the anti-bacterial activity. The number of active hydroxyl groups in the nanotube structure could greatly enhance the ‘adsorption’ of the bacterial cell, and thereby enhanced antibacterial properties can be achieved (29).

## Conclusion

Based on our results, it can be concluded that the modification of denture base acrylic resin with TiO_2_ nanotubes can greatly improve its antimicrobial properties. Hence, the novel PMMA/ TNTs composite can be considered as a promising material for fabrication of acrylic resin base dental materials. However, further studies have to be done to evaluate the biological response of oral tissues to PMMA/ TiO_2_ nanotube composites.
